# Should I Stay, or Should I quit? The Effect of Bullying, Emotional Exhaustion and Work Engagement in Public Healthcare Workers: A Latent Profile Analysis

**DOI:** 10.17505/jpor.2025.28313

**Published:** 2025-10-02

**Authors:** Francesco Buscema, Laura Lorente Prieto, Daniela Converso, Lara Colombo

**Affiliations:** 1 Department of Psychology, University of Turin, Turin, Italy; 2 IDOCAL, University of Valencia, Valencia, Spain

**Keywords:** bullying, latent profile analysis, person-centered approach, emotional exhaustion, work engagement

## Abstract

The aim of this study was to identify profiles of workplace bullying, emotional exhaustion, and work engagement in a sample of public healthcare workers in Italy. Drawing on the Job Demands-Resources Model, we explored how job demands (quantitative demands, perceived injustice, and role conflict), job resources (quality of the work environment, career development, horizontal and vertical trust, social support), and personal resources (self-efficacy, and passion for work), predict these profiles. In addition, we examined how the identified profiles relate to organisational and personal outcomes (physical and psychological symptoms, intention to quit, performance, and job satisfaction). 624 healthcare workers completed an online survey and latent profiles analysis were performed to identify profiles. Latent factor analysis and multinomial logistic regression analysis were performed. Results allowed the identification of four profiles (*Balanced, Engaged, Bullied Not Stressed*, and *Bullied & Stressed*), which differed significantly in job demands, resources and outcomes. Our findings highlight the complexity of public healthcare workers’ experiences and provide evidence for strategic interventions aimed at optimising working conditions to enhance both employee well-being and organisational effectiveness. Understanding workplace experiences through a person-centered lens allows for more tailored strategies to support staff well-being and performance in high-pressure environments such as public healthcare.

## Introduction

Working in healthcare often involves high levels of *emotional exhaustion* (EE) due to various factors such as the difficult demands of patients and users and the high workload, but also strong *work engagement* (WE) due to patient life activities (Dal Santo et al., 2024; Sarabia-Alcocer et al., 2024). Recent studies show that social support from colleagues plays a crucial role in promoting WE and helps to manage EE (Cabana-Mamani et al., 2024; Dal Santo et al., 2024). Unfortunately, social support from colleagues is not always guaranteed, and the prevalence of *workplace bullying* (WB) can severely affect both emotional well-being and WE (Liang, 2021).

However, the relationship between EE, WE and WB has been little studied with a person-centered approach in healthcare workers (HW). This study enriches the knowledge in this field by identifying profiles of EE, WE and WB in Italian HW and by examining how the co-occurrence of EE, WE and WB is predicted by job demands and job resources. Furthermore, this study explores how these profiles are related to personal and organisational outcomes such as intention to quit, health symptoms, job performance and job satisfaction.

### The Indicators of Latent Profiles: EE, WE, and WB

Assessing EE, WE and WB in HW is crucial as these factors have a significant impact on employee well-being and performance. EE in public HW is a multifaceted issue that is exacerbated by numerous factors such as workplace demands and organisational conditions. In various theories, EE is often referred to as a core dimension of job burnout (Maslach & Leiter, 2016; Schaufeli et al., 2020). Research shows that the unique environment of public health, characterised by bureaucratic pressure and limited resources, contributes significantly to increased levels of EE among employees (López-Cabarcos et al., 2021; Opoku et al., 2021).

Combined with EE, WB is a critical problem that negatively impacts HW. WB can manifest in various forms, including verbal abuse, excessive work demands, and social undermining, resulting in a toxic work environment (Ribeiro & Sani, 2024). Research suggests that WB exacerbates EE and decreases job satisfaction and WE among HW (Cao et al., 2023; Ribeiro & Sani, 2024). This link between WB, EE and WE emphasises the need for comprehensive workplace policies that combat WB while promoting a culture of respect and compassion among employees.

Assessing WE is equally important as it serves as a protective factor against burnout. WE characterised by vigour, dedication and absorption (Bakker & Schaufeli, 2015), can act as a buffer against EE (Mazzetti et al., 2023). However, if the work environment is characterised by WB and excessive demands, the sense of commitment can diminish, leading to a cycle of dissatisfaction and burnout (Wang et al., 2022).

In critical times such as the COVID-19 pandemic, the interplay of these factors is even more pronounced. HW faced unprecedented demands that simultaneously increased their risk of EE and exposed them to WB behaviour that was exacerbated by societal stressors (d’Ettorre et al., 2021). Furthermore, EE during this time has been linked to lower job satisfaction and WE, reinforcing a cycle of declining morale and productivity (Sexton et al., 2022).

The consequences of EE extend beyond the individual, affecting organisational performance and the quality of patient care (Opoku et al., 2021). Furthermore, public HW reported higher levels of EE compared to their counterparts in the private sector, largely due to the debilitating effects of bureaucratic stressors in their work environment (Simonovich et al., 2022). The need for targeted interventions to combat EE and WB is further compounded by the particular vulnerability of certain subgroups within the healthcare professions, such as nurses and frontline workers (Ribeiro & Sani, 2024).

### Predictors of the Latent Profiles: Job Demands and Job Resources

Job demands are critical components that affect the psychological and emotional state of HW. Quantitative demands related to work volume and time pressure have been shown to correlate with feelings of being overwhelmed and EE among HW (Barello et al., 2021). In addition, role conflict, another aspect of job demands, significantly exacerbates stress as workers are expected to fulfil multiple objectives that may conflict with each other, leading to increased frustration and feelings of inadequacy (Gynning et al., 2024). Perceived injustice also plays a central role in HW affecting emotional well-being and WE, leading to lower job satisfaction (Thapa et al., 2022).

Conversely, job resources serve as an essential buffer against these demands and their potentially harmful effects. The quality of the work environment has been shown to improve workers’ feelings of safety and support, which can significantly increase job satisfaction and WE (Kohnen et al., 2024). In addition, horizontal and vertical trust are key factors that contribute to the emotional resilience of HW (Jimoh, 2021). Social support, either through collegial relationships or support from supervisors, has been repeatedly cited as a protective factor against burnout, emphasising the need for supportive networks within the healthcare work environment (Huang et al., 2022; Mori et al., 2022).

In terms of personal resources, self-efficacy and passion at work are important professional resources that directly influence both WE and EE. Self-efficacy has been associated with proactive coping strategies and improved performance metrics in HW, which mitigate the effects of high job demands (Looi et al., 2024). In addition, passion for work fosters WE and serves as a motivating force that encourages individuals to effectively overcome challenges and increase overall job satisfaction (van Engen et al., 2022).

### Personal and Organizational Outcomes of the Latent Profiles

WB, EE and WE among HW have been studied extensively due to their negative impact on personal and organisational outcomes (Ariza-Montes et al., 2013; Cao et al., 2023; Ul Haq & Huo, 2024). Studies suggest that healthcare professionals who suffer from WB are more likely to suffer from depression, anxiety and somatic complaints leading to physical symptoms such as fatigue and headaches (ArizaMontes et al., 2013; Ul Haq & Huo, 2024). In addition, the constant stress caused by WB contributes to EE, which affects both physical abilities and mental resilience and ultimately impairs general well-being (Ul Haq & Huo, 2024). Research shows that HW who experience high levels of EE report lower job satisfaction and an increase in psychological distress, which is detrimental to their mental health (Gedik et al., 2023; van Elk et al., 2023).

Moving from individual outcomes to the organisational perspective, the impact of EE and WB on intention to quit and job performance becomes increasingly clear. Research shows that EE serves as a mediator between WB and intention to quit (Gedik et al., 2023). HW who experience WB may no longer feel validated in their role, leading them to consider leaving their job (Gedik et al., 2023). In terms of work performance, there is evidence that when HW are emotionally exhausted, WE decreases, resulting in lower patient satisfaction and lower overall performance metrics (Fiabane et al., 2019; Gedik et al., 2023; Ul Haq & Huo, 2024). In addition, improving the work environment to reduce WB and promote emotional well-being could increase employee performance, WE and retention (Sarfraz et al., 2022). Studies show that employees who experience high levels of WB also report low job satisfaction (Cao et al., 2023; Fiabane et al., 2019). The psychological impact of WB can affect the sense of belonging among colleagues, which reduces overall job satisfaction. In addition, a negative psychosocial climate in the workplace contributes to an environment in which employees feel undervalued and overwhelmed, which exacerbates EE (Huseynova & İslamoğlu, 2024). When healthcare organisations prioritise interventions to reduce WB and promote emotional health, they can significantly increase employee satisfaction, increase employee retention and improve organisational performance (Samad Dahri et al., 2020).

### Overview of the Present Study

The present study aimed to identify profiles of WB, EE and WE among Italian public HW using latent profile analysis (LPA) and to examine their relationships with predictors (i.e., job demands, job resources and personal resources), and with organisational and personal outcomes. The theoretical framework for our study is the JD-R model (Bakker et al., 2023). This model states that work stress can be the result of an imbalance between the demands of workplaces on the individual and the resources available to the individual to cope with job demands (Bakker et al., 2023). In this sense, each work context has specific risk factors that can be grouped into two general categories: job demands and job resources. Job demands are the physical, psychological, social and organisational aspects of work that require the mobilisation of energy in the form of effort or use of skills, and which impose costs on the individual at a physical and psychological level. On the other hand, job resources are those physical, psychological, social and organisational aspects that are a functional tool for achieving work goals, that help to cope with job demands or contain the associated psychological costs, and that stimulate and support personal growth and development (Bakker et al., 2023). We chose the JD-R model because it assesses both job demands and resources and interprets the impact of personal and organisational outcomes, taking into account the interdependencies between these factors (Bakker et al., 2023).

Based on previous research (Franzoi et al., 2021; Sardella et al., 2024; Ul Haq & Huo, 2024), we expect WB, WE and EE scores in HW to be summarised in several profiles that represent an overall quality of the psychological work environment as a multidimensional concept. We hypothesised that the first latent profile would be close to the average on all dimensions included and thus could be described as a “balanced” profile (Hypothesis 1a). We expected to find a second profile characterised by high values for negative actions and EE and low values for WE that could be referred to as “Bullied & Stressed” (Hypothesis 1b). Third, we expected a profile characterised by high scores on negative actions but low scores on WE and EE that could be referred to as “Bullied without stress” (Hypothesis 1c). Finally, we also expected to find a fourth profile with high WE and low scores on negative actions and EE that could be labelled “Engaged” (Hypothesis 1d).

We followed the recommendations of Schaufeli and Bakker (2004), who consider EE as a core dimension of burnout and WE as an independent construct, in contrast to Maslach and Leiter (2016), who consider burnout risk and WE as two endpoints of the burnout risk continuum. In addition, recent studies indicated a direction in the pattern of job demands and resources in HW (Franzoi et al., 2021; Sardella et al., 2024; Ul Haq & Huo, 2024). According to the JD-R model, participants with low job demands and high job resources should be categorised in the “Engaged” profile (Hypothesis 2). For the personal and organisational outcomes, we assumed significant differences between all profiles (Hypothesis 3) and that the “Engaged” profile has higher mean values for job performance and job satisfaction and lower mean values for physical and psychological symptoms and intention to quit compared to all other profiles.

## Methods

### Participants and Procedure

Participants were recruited from a metropolitan National Health Service (NHS) in northern Italy. The number of employees of the NHS is around six thousand healthcare workers. To achieve a representative sample, the health and safety department of the organization sent to all the employees an email including the link of the informed consent of the questionnaire. All participants gave informed consent. Following their consent, an online survey was distributed to participants via the open-source platform LimeSurvey from December 2024 to February 2025. A total of 843 employees gave the informed consent to the survey, but only 624 HW completed the questionnaire and were included in the analysis. Of the participants, 482 (77.2%) were female, 135 (21.6%) male and 7 (1.1%) no-binary. Most participants were between 48 and 58 years old (39.9%) and worked as clinicians (76.9%). The demographic characteristics are shown in more detail in Table 1.

**Table 1:** Demographic characteristics of participants (N = 624)

Variables	N	%
Gender		
Male	135	21.6
Female	482	77.2
Non-binary	7	1.1
Age		
≤ 25 y	11	1.8
26 – 36 y	141	22.6
37 – 47 y	120	19.2
48 – 58 y	249	39.9
≥ 59 y	103	16.5
Work activity		
Clinical ^a^	480	76.6
Administrative	83	13.3
Technical	48	7.7
Other	13	2.1
Marriage		
Unmarried	164	26.3
Married	381	61.1
Divorced	76	12.2
Widow/er	3	0.5
Expertise		
≤ 9 y	157	25.2
10 – 21 y	163	26.1
22 – 30 y	168	26.9
≥ 31 y	136	21.8
Job hierarchy		
No responsibilities	545	87.3
Middle management	66	10.6
High management	13	2.1

a This category includes physicians, nurses, and all the healthcare professionals related to taking care of patients.

## Measures

### The latent profile indicators

The latent profile indicators included the dimensions *workplace bullying* (WB), *work engagement* (WE) and *emotional exhaustion* (EE). WB was measured using the Italian version of the Short Negative Acts Questionnaire (S-NAQ) (Balducci et al., 2010). EE was measured using the short version of the relative subscale from the Italian version of the Burnout Assessment Tool (BAT) (Mazzetti et al., 2023; Schaufeli et al., 2020). Studies confirm that this subscale effectively reflects the core characteristics of burnout, emphasising its relevance to emotional states faced by HW (Fiabane et al., 2019; Innstrand, 2022). To measure WE, we used the Italian version of the Super-Short Utrecht WE Scale (Schaufeli et al., 2019).

### The predictors

The predictors were divided into job demands, job resources and personal resources. Job demands included in the study are quantitative demands, perceived injustice, and role conflict. *Quantitative demands* were measured using the Quantitative Workload Inventory (Spector & Jex, 2011) and refer to the perceived workload in terms of quantity and intensity. This dimension makes it possible to determine the individual perception of the amount of work the HW has to cope with. *Perceived injustice* was measured using the Majer D’Amato Organisational Questionnaire (Majer & D’Amato, 2001) describes the participants’ feeling of unfairness in relation to the organisation's decisions towards employees. *Role conflict* was measured using a five-point Liker scale adaptation of the Copenhagen Psychosocial Questionnaire (COPSOQ III) (Burr et al., 2019) to achieve a better fit with the rest of the questionnaire and refers to the degree of clarity and contradiction in job requirements.

Job resources include the quality of the work environment, career development, horizontal and vertical trust, and social support, while personal resources include self-efficacy at work, and passion at work. *The quality of the work environmen*t refers to the basic needs that an employee should receive from the company at work, such as tools, enough colleagues to fulfil the tasks and training for the work activities and was measured using the job satisfaction scale from the second version of the Copenhagen Psychosocial Questionnaire (Pejtersen et al., 2010). *Career development* was measured using a five-point Liker scale adaptation of the “development opportunities” subscale of the COPSOQ III (Burr et al., 2019), which refers to the participants’ belief that they have opportunities to make career moves. *Horizontal and vertical trust* was measured using a five-point Liker scale adaptation of subscales of COPSOQ III (Burr et al., 2019) that refer to the adequacy of the processes that ensure the access and dissemination of information, as well as the way in which all organisational components are involved in the work processes and the trust that exists between them. *Social support* was measured using a five-point Liker scale adaptation of two subscales of the COPSOQ III (Burr et al., 2019) respectively called “Social support from supervisor” and “Social support from colleagues” that refers to aspects of relationships among peers and with direct supervisors that can affect well-being at work.

*Self-efficacy* at work was measured using the self-efficacy subscale from the COPSOQ III (Burr et al., 2019), which refers to the feeling of competence and effectiveness in relation to the participants’ work. *Passion at work* was measured using the “harmonious passion” subscale from the Italian version of the Passion for Work Scale (Zito & Colombo, 2017) that refers to the sense of appreciation that employees feel when they think about their work.

### The outcomes

The personal and organisational outcomes included the following dimensions: physical symptoms, psychological symptoms, intention to quit, performance and job satisfaction. *Physical symptoms* and *psychological symptoms* were measured to assess the health impairments – both physical and psychological – of HW; to be consistent with the instruments used, we adopted the two subscales of the BAT (Mazzetti et al., 2023). *Intention to quit* was measured using and adaptation of Cortese’s scale (2013) and indicates how often the participant has thought about changing jobs or requesting a transfer in the last six months. *Performance* was measured using the scale of Olvera-Calderón et al. (2017) which assess group performance in two factors: extra-role and intra-role. *Job satisfaction* was measured with a single tailored item that asked how satisfied the employee was with their job. A list of all the variables included in the study is provided in Appendix A.

### Data Analysis

Firstly, descriptive analyses, correlations and reliability analyses were performed with all the study variables. Secondly, latent profile analysis (LPA) with MPlus and multinomial logistic regression analysis with SPSS were performed to answer the research question. The LPA included the following dimensions as observed indicators: EE, WE and WB. We analysed the fit indices of the measurement models by starting with one profile and gradually adding classes. We stopped estimating additional profiles when empirical under-identification or convergence problems occurred. We selected the best fitting models based on absolute and relative fit indices (Sorgente et al., 2019). Both the likelihood ratio chi-square of the goodness of fit $\left(\chi_{LRT}^{2}\right)$ and the standardised residuals for each response pattern can be used as a measure of the absolute model fit. Five information criteria (IC) can be used as descriptive measures for the relative model fit: the Akaike information criterion (AIC), the Consistent Akaike Information Criterion (CAIC), the Approximate Weight of Evidence (AWE), the Bayesian Information Criterion (BIC), and the Samplesize Adjusted Bayesian Information Criterion (SS-BIC). Smaller IC values indicate a better fit, while the BIC is useful to estimate the Schwarz Information Criterion (SIC) (Sorgente et al., 2019). The Bayes factor (BF) compares two models each (*k* and *k*+1 model) and the best model is the simplest *k*-class model with BF > 3. The approximate probability of a correct model probability (cmP) compares all candidate models - any model with cmP > .10 can be considered a candidate model (Sorgente et al., 2019). The statistical tests that can be used as inferential measures of relative fit indices are the Vuong-Lo-Mendell-Rubin likelihood ratio test (VLMR-LRT), the adjusted Lo-Mendell-Rubin likelihood ratio test (adj. LMR-LRT) and the parametric bootstrapped likelihood ratio test (BLRT). For these three tests a statistically significant p-value indicates that the *k*-class model fits the data significantly better than a model with one less class (Sorgente et al., 2019). The most common diagnostic classification is entropy (Ek). Higher Ek values signal better classification clarity, with an established anecdotal threshold of 0.80 often cited as a measure of optimal model fit (Lonigan et al., 2018; Zhang et al., 2023). However, several researchers have pointed out that some studies have successfully used models with lower entropy values, including those in the 0.70 to 0.79 range, effectively strengthening the results despite a suboptimal entropy score (McDermott et al., 2022; Sandborg et al., 2022). Furthermore, the quality of the classification is evaluated by checking the class proportion (CP), the modal class assignment proportion (mcaP), average posterior probability (avePP), and odds of correct classification (OCC). Classification can be considered good if the mcaP for each profile is included in the 95% CI of the CP, avePP values are equal to .70 or higher and the OCC values are above 5 (Masyn, 2013; Sorgente et al., 2019). After finding the best LPA model, we stored the most likely profile membership for each person with an observed variable representing each participant’s membership in the well-being and work effectiveness groups. Then, chi-square test was performed to analyse the statistical differences between the socio-demographic variables (i.e., gender, age, work activity, marriage, expertise, and job hierarchy) and the profiles. Nominal logistic regression models were then run to describe the profiles in relation to the job demands variables and job resources. Finally, we conducted a series of one-way analyses of variance (ANOVA) to test whether the profiles were related to the outcomes. Post hoc analyses were conducted using the Bonferroni test.

## Results

### Preliminary Analyses

The mean values, standard deviation and correlations of the main variables are listed as supplementary material (Appendix B). The correlations between the indicators, the predictors and the outcomes are all significant and in the expected sense. This shows that both WB and EE are positively related to job demands and negatively to outcomes (i.e. physical and psychological symptoms and intention to quit), while they are negatively related to job resources and positively to outcomes (i.e., performance and job satisfaction). In contrast, WE are negatively related to job demands and negative outcomes, while positively related to job resources and positive outcomes.

The internal consistencies of all measured variables are shown in Table 2 using McDonald’s Omega.

**Table 2:** Internal consistency of all the variables included in the study (N = 624)

	Items	Ω
Bullying	9	.92
Emotional exhaustion	3	.89
Work engagement	3	.79
Quantitative demands	5	.90
Perceived injustice	4	.87
Role conflict	3	.84
Quality of work environment	4	.76
Career development	3	.89
Horizontal and vertical trust	4	.82
Social support	3	.70
Self-efficacy at work	4	.81
Passion at work	6	.90
Physical symptoms	5	.77
Psychological symptoms	5	.83
Intention to quit	3	.77
Job satisfaction	1	--

*Note*: When number of items is less than three McDonald’s Omega can’t be calculated.

### Identification of the Latent Profiles

**Figure 1:**
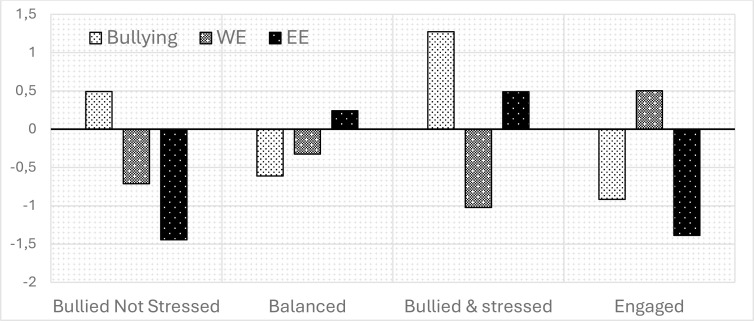
Mean scores and labels of the 4-latent profiles solution.

We estimated ten different models of latent profiles; we did not estimate the 11-class model because its best loglikelihood was not replicated. As shown in Table 3, the 4-profile and 7-profile solutions had satisfactory goodness-of-fit indices. Therefore, the 4-profile, 5-profile and 7-profile solutions were analysed by classification diagnostics. The 10-profile solution was excluded from the classification diagnosis due to the lower number of cases for the profile. As shown in Table 4, all solutions met the classification diagnostic criteria. Consequently, we interpreted the classes obtained in all solutions and decided to keep the 4-profile solution, as it shows a profile (*Balanced*) that is relevant from a theoretical point of view and disappears in the next solutions. The mean of the standardised score of each profile of the 4-profile solution is shown in Figure 1.

**Table 3:** Comparing models fit for different well-being and work effectiveness.

Model	LL	*df*	AIC	CAIC	AWE	BIC	SS-BIC	BF	cmP	VLMRT (*p*)	LMR-LRT (*p*)	BLRT (*p*)	Entropy
1 profile	-2.654.752	6	5321.504	5354.120902	5404.737804	5348.120902	5329.071779	0.00	1.55E-64				
2 profiles	-2.559.126	10	5138.252	5192.613504	5276.975007	5182.613504	5150.864965	0.00	1.34836E-28	p < .0001	p < .0001	p < .0001	0.784
3 profiles	-2505.265	14	5038.53	5114.636105	**5232.74221**	5100.636105	5056.188152	0.00	8.53047E-11	p < .0001	p < .0001	p < .0001	0.703
4 profiles	-2478.855	18	4993.71	5091.560707	5243.411413	5073.560707	5016.413338	0.00	6.46132E-05	**0.0212**	**0.0239**	**p < .0001**	0.774
5 profiles	-2457.725	22	4959.45	**5079.045308**	5264.640616	5057.045308	4987.198524	**4.70**	**0.249226561**	0.0527	0.0578	p < .0001	0.766
6 profiles	-2446.4	26	4944.8	5086.13991	5305.479819	5060.13991	4977.59371	0.08	0.053040811	0.2903	0.2994	p < .0001	0.748
7 profiles	-2430.981	30	4921.962	5085.046511	5338.131022	**5055.046511**	4959.800896	**132.07**	**0.677060675**	0.0894	0.0955	p < .0001	0.779
8 profiles	-2422.992	34	4913.984	5098.813113	5385.642225	5064.813113	4956.868082	0.34	0.00512669	0.4637	0.4751	0.04	0.788
9 profiles	-2409.027	38	4894.054	5100.627714	5421.201428	5062.627714	4941.983269	**79.94**	0.02	0.2484	0.2623	p < .0001	0.804
10 profiles	**-2400.536**	42	**4885.072**	5113.390315	5467.708631	5071.390315	**4938.046455**	0.00	0.00	0.5118	0.5204	p < .0001	0.818

**Table 4:** Classification diagnostic criteria.

Model	Entropy	Profile (N)	CP	95% CI	mcaP	AvePP	OCC
4-profiles	0.774	Profile 1 (n=51)	0.088	(0.039 0.261)	0.08173	0.799	41.20
		Profile 2 (n=317)	0.493	(0.409 0.573)	0.50801	0.884	7.84
		Profile 3 (n=95)	0.157	(0.110 0.212)	0.15224	0.881	39.75
		Profile 4 (n=161)	0.262	(0.195 0.321)	0.25801	0.877	20.08
5-profiles	0.766	Profile 1 (n=69)	0.115	(0.081 0.151)	0.11058	0.930	102.24
		Profile 2 (n=176)	0.268	(0.199 0.325)	0.28205	0.812	11.80
		Profile 3 (n=154)	0.244	(0.172 0.307)	0.24679	0.866	20.02
		Profile 4 (n=199)	0.326	(0.252 0.428)	0.31891	0.838	10.69
		Profile 1 (n=26)	0.047	(0.014 0.094)	0.04167	0.872	138.13
7-profiles	0.779	Profile 1 (n=166)	0.255	(0.199 0.318)	0.26603	0.821	13.40
		Profile 2 (n=59)	0.095	(0.043 0.131)	0.09455	0.764	30.84
		Profile 3 (n=22)	0.032	(0.008 0.062)	0.03526	0.835	153.08
		Profile 4 (n=120)	0.186	(0.110 0.234)	0.19231	0.776	15.16
		Profile 5 (n=56)	0.097	(0.058 0.138)	0.08974	0.871	62.86
		Profile 6 (n=68)	0.112	(0.085 0.145)	0.10897	0.934	112.20
		Profile 7 (n=133)	0.223	(0.167 0.308)	0.21314	0.847	19.29

A total of 51 participants (8.2%) were classified into the first profile, labelled *Bullied Not Stressed*, characterised by the absence of EE, below average WE scores and the presence of WB, and. 317 participants (50.8%) were classified into the second profile, labelled *Balanced*, characterised by scores below average for WB and scores closer to average for both WE and EE. 95 participants (15.2%) were assigned to the third profile, labelled *Bullied & Stressed*, characterised by higher scores for WB, lack of WE and high scores for EE. 161 participants (25.8%) were classified to the last profile, labelled *Engaged*, characterised by the absence of WB and EE and high levels of WE.

### Differences Between Profiles in Job Demands, Resources and Outcomes

We examined whether the job demands, job resources and sociodemographic had a significant association with the profiles using multinomial logistic regression analysis and chi-square test. There are no significant differences between all the sociodemographic variables (i.e., gender, age, work activity, marriage, expertise, and job hierarchy) and the latent profiles. We used the *Engaged* profile as preference group for the multinominal logistic regressions with job demands and job resources variables (Tables 5 and 6).

**Table 5:** Multinomial logistic regression coefficients for latent profiles with Job Demands variables with the “Engaged” profile as the reference group (R2STEP).

Predictor	*Balanced*				*Bullied Not Stressed*				*Bullied & Stressed*			
	Coeff.	*SE*	*p*	*OR*	Coeff.	*SE*	*p*	*OR*	Coeff.	*SE*	*p*	*OR*
Quantitative demands	.663	.126	**<.001**	1.941	-.243	.175	.165	.785	.603	.180	**<.001**	1.828
Perceived injustice	.369	.126	**.003**	1.447	.783	.783	**<.001**	2.189	.795	.175	**<.001**	2.215
Role conflict	.640	.135	**<.001**	1.897	1.039	.204	**<.001**	2.827	1.663	.220	**<.001**	5.273

**Table 6:** Multinomial logistic regression coefficients for latent profiles with Job Resources variables with the “Engaged” profile as the reference group (R2STEP).

Predictor	*Balanced*				*Bullied Not Stressed*				*Bullied & Stressed*			
	Coeff.	*SE*	*p*	*OR*	Coeff.	*SE*	*p*	*OR*	Coeff.	*SE*	*p*	*OR*
Quality of work environment	-.189	.130	.146	.827	-.354	.209	.090	1.424	-.008	.179	.966	.992
Career development	-.156	.149	.293	.855	-.587	.216	.006	.556	-.190	.193	.325	.827
Horizontal and vertical trust	-.419	.156	**.007**	.658	-.890	.252	**<.001**	.411	-.966	.217	**<.001**	.381
Social support	-.377	.163	**.021**	.686	-.829	.230	**<.001**	.437	-.957	.202	**<.001**	.384
Self-efficacy at work	-.167	.132	.207	.846	-.366	.202	.069	.693	-.134	.173	.437	.874
Passion at work	-.725	.157	**<.001**	.484	-.221	.239	.355	.802	-.711	.209	**<.001**	.491

The multinomial logistic regression for latent profiles with job demands shows that HW experiencing more quantitative demands (*OR* = 1.94), perceived injustice (*OR* = 1.45), and role conflict (*OR* = 1.90) were more likely to be categorized in the *Balanced* profile compared to the *Engaged* profile. HW experiencing more perceived injustice (*OR* = 2.19) and role conflict (*OR* = 2.83) were more likely to be categorized in the *Bullied Not Stressed* profile compared to the *Engaged* profile, while there were no significant difference between both profiles referring to quantitative demands. The HW experiencing more quantitative demands (*OR* = 1.83), perceived injustice (*OR* = 2.21), and role conflict (*OR* = 5.27) were more likely to be categorized in the *Balanced* profile compared to the *Engaged* profile.

The multinominal logistic regression for latent profiles with job resources shows that HW with lower social support (*OR* = 0.69), horizontal and vertical trust (*OR* = 0.66), and passion at work (*OR* = 0.49) were more likely to be categorized in the *Balanced* profile compared to the *Engaged* profile. There are no significant differences between both profiles referring to quality of work environment, career development, and self-efficacy at work. HW experiencing less horizonal and vertical trust (*OR* = 0.41) and social support (*OR* = 0.44) are more likely to be categorized in the *Bullied Not Stressed* profile than the *Engaged* profile. There are no significant differences between both profiles referring to all the other job resources. Finally, the HW that experience less horizontal and vertical trust (*OR* = 0.38), social support (*OR* = 0.38), and passion at work (*OR* = 0.49) are more likely to be categorized in the *Bullied & Stressed* profile than the *Engaged* profile. There are no significant differences between both profiles referring to all the other variables.

In conclusion, we investigated whether the four profiles differed with respect to organizational (i.e. performance and job satisfaction) and personal outcomes (i.e. physical symptoms, psychological symptoms, and intention to quit) using one-way ANOVA. Our results revealed significant difference among the profiles, as displayed in Table 7. Post hoc comparisons using *t*-test with Bonferroni correction indicated that the *Engaged* profile reported lower scores on physical and psychological symptoms and intention to quit, and higher scores on performances and job satisfaction (*p*s < 0.001) compared with all the other profiles. The *Bullied & Stressed* profile presented the highest scores on physical and psychological symptoms, intention to quit, and the lowest scores on performances and job satisfaction (*p*s < 0.05) compared with all the other profiles. However, almost all the other profiles are statistically different when related with outcomes variables, except for the *Balanced* profile and the *Bullied Not Stressed* on physical symptoms, intention to quit, and job satisfaction, and the *Bullied Not Stressed* profile and the *Bullied & Stressed* profile on performance.

**Table 7:** Means, standard deviations. and one-way analyses of variance in personal outcomes.

Measure	*Balanced*		*Bullied & Stressed*		*Bullied Not Stressed*		*Engaged*		*F*(3, 620)	*η^2^*
	*M*	*SD*	*M*	*SD*	*M*	*SD*	*M*	*SD*		
Physical symptoms	**.17**	.92	**.66**	1.03	**.02**	1.01	**-.72**	.68	56.36***	.21
Psychological symptoms	**.27**	.81	**.80**	.84	**-.22**	.93	**-.94**	.70	120.04***	.37
Intention to quit ^a^	.22	.92	.60	.83	.13	.87	-.82	.76	70.71***	.26
Performance ^a^	.00	.91	-.58	1.19	-.41	1.07	.48	.75	29.24***	.12
Job satisfaction	-.17	.90	-.73	1.02	-.16	.92	.82	.60	76.45***	.27

** *p* < 0.001.

a *F*-statistic from ANOVA is calculated for a reduced sample of 617 participants.

## Discussion

The present study aimed to identify profiles of WB, EE and WE in a sample of 624 public healthcare workers in Italy. In support of hypothesis 1, four profiles were identified in this study: *Balanced, Engaged, Bullied Not Stressed* and *Bullied & Stressed*). Half of the sample of HW showed a *Balanced* profile (50.8%), having scores close to the mean of the indicators scale and a quarter of the sample having high WE score. A quarter of the HW sample showed an *Engaged* profile (25.8%), while the remaining two profiles both had high scores on WB, but one of them also had higher scores on EE (15.2%), while the other scored low on EE (8.2%).

The *Balanced* profile reflects the typical work environment, struggles and challenges of public health workers (Acquadro Maran et al., 2023; Fattori et al., 2022). The presence of EE, which is slightly above the average of the scale, is compensated by the slightly reduced presence of WE, which also shows the typical situation in public health, with participants who are exhausted due to job demands, while resources are not fully able to compensate for the impact of demands, which affects the reduction in WE (Ribeiro & Sani, 2024). This profile had low levels of WB, suggesting that the situation regarding this issue is not risky.

The *Engaged* profile can be understood within the framework highlighting both individual motivational factors and a supportive work environment, as high levels of WE are typically associated with robust job resources, a positive psychosocial safety climate and organisational practises that strengthen personal empowerment and resilience (Dennerlein et al., 2020).

In the *Bullied Not Stressed* and *Bullied & Stressed* profiles, WB appears as a common denominator that undermines intrinsic motivation and reduces WE, regardless of other stress indicators (Acquadro Maran et al., 2023). In particular, the *Bullied Not Stressed* profile suggests that although the work environment is affected by WB, some employees benefit from alternative coping strategies, robust personal resilience or a degree of supportive social capital that buffers the impact on their emotional state (Ribeiro & Sani, 2024). In contrast, in the *Bullied & Stressed* profile the co-occurrence of high WB and high EE reflects a dangerous imbalance between job demands and job resources and emphasises the need for targeted interventions that focus on both reducing WB and strengthening support systems to mitigate the stress outcomes (Fattori et al., 2022; Ribeiro & Sani, 2024).

Regarding hypothesis 2, we can conclude that our results support it since HW who experience lower quantitative demands, less role conflict and a stronger perception of impartial treatment are more likely to be classified as showing an Engaged profile. When job demands such as heavy workloads, conflicting role expectations and perceived unfair treatment are minimised, employees can devote more personal resources to meaningful WE rather than spending energy on coping with stress (Bakker et al., 2023; Fattori et al., 2022). This understanding is informed by the JD-R model, which highlights the importance of job resources in enhancing job satisfaction and WE among HW.

Results also show complex relationships between job resources, WB and EE (Fattori et al., 2022). A comparison of the *Balanced* and *Engaged* profiles shows that while some structural or tangible resources such as the quality of the work environment, career development and self-efficacy may be sufficient – as no significant differences were found on these variables – the lack of robust interpersonal resources plays a crucial role in distinguishing employees with high WE. The diminished interpersonal resources may hinder motivational processes that are essential for maintaining WE, thus predisposing employees to experience a more balanced but less energised work state (Amiri et al., 2024).

When the *Bullied Not Stressed* and the *Engaged* profiles are compared, it is plausible to conclude that while the structural aspects of the workplace remain intact, deficits in trust and social support make individuals more vulnerable to WB incidents, even if these experiences do not directly translate into increased stress levels (Ribeiro & Sani, 2024). This decoupling suggests that some employees may be able to withstand the initial stress of WB due to other coping mechanisms, but the lack of important relational job resources remains a significant risk factor for negative psychosocial outcomes when WB occurs. Finally, when comparing the *Bullied & Stressed* profile with the *Engaged* profile, the combined lack of relational (trust and social support) and motivational (passion at work) resources appears to exacerbate the effects of WB and lead to an accumulation of stress. The lack of significant differences in job resources emphasises that interpersonal dynamics and emotional engagement are critical in buffering or contributing to the consequences of WB (Amiri et al., 2024).

Finally, our results largely support hypothesis 3, showing differences between profiles. The results show that the four latent profiles differ significantly in both organisational outcomes (performance and job satisfaction) and personal outcomes (physical symptoms, psychological symptoms, and intention to quit). In particular, the *Engaged* profile is associated with the most favourable outcomes – characterised by higher performance and job satisfaction as well as lower physical and psychological symptoms and a lower intention to quit. These results suggest that WE is a protective factor that supports both high organisational productivity and positive individual health outcomes. In contrast, the *Bullied & Stressed* profile shows the worst results in both groups of measurements. Specifically, individuals in this profile report the highest physical and psychological symptoms, the highest intention to quit, and the lowest performance and job satisfaction, suggesting that the combination of WB and high stress impairs both job performance and overall well-being (D’Alleva et al., 2023; Marin et al., 2024).

It is also noteworthy that when comparing the *Balanced* and *Bullied Not Stressed* groups, there are no significant differences in terms of physical symptoms, intention to quit and job satisfaction. This suggests that while these two profiles have similar scores on some negative outcomes, the absence of high stress in the *Bullied Not Stressed* group is not sufficient to produce differences in these specific personal outcomes compared to the *Balanced* group. Similarly, the lack of significant differences in performance between the *Bullied Not Stressed* and *Bullied & Stressed* profiles suggests that while WB may affect performance, the additional EE exacerbates other personal health outcomes rather than further affecting performance alone. These nuances reflect the intertwined nature of interpersonal conflict and stress responses in the workplace, where the presence of both factors can lead to increased negative effects on organisational health and productivity (D’Alleva et al., 2023).

These findings are consistent with previous research indicating robust associations between workplace conditions – particularly the presence of WB and EE – and negative outcomes such as burnout, deteriorated mental and physical health, reduced job satisfaction and lower work performance (Marin et al., 2024). From an applied perspective, the findings highlight the importance of fostering an engaging work environment in healthcare contexts by introducing supportive organisational practises and mitigating negative behaviours in the workplace.

### Limitations of the Present Study

We should note several limitations of our research and how we addressed them. First, our study had a cross-sectional design, which limits the conclusions that can be drawn. A longitudinal study could have revealed changes over time, eliminating cohort effects and providing more information on the evolution of the participants within each identified latent profile.

Second, mostly of the participants were female; however this can also be seen as a strength, particularly in the healthcare sector where women represent most of the workforce. So, our study also contributes valuable insights to the growing body of gender-sensitive occupational research and supports the development of interventions that are more tailored and effective for female employees.

Third, the results of this study were found within a big company working in Italian NHS. Future studies could try to replicate these findings in other countries.

Finally, LPA was used in the classification analysis because of its valuable model-based properties, which are lacking in alternative more descriptive methods of classification. However, a control analysis using a standard cluster analytic algorithm produced a classification structure that differed from the one produced by the LPA analysis. This suggests that the types found should be regarded as tentative.

### Implications of the Present Study

The present study carries significant implications for both theory and practice in the healthcare sector. From a theoretical standpoint, by extending the JD-R model through a person-centered approach, the study contributes to a more nuanced understanding of how distinct latent profiles of WB, EE, and WE manifest among Italian public HW. Such differentiation between profiles underscores the complexity of workplace experiences and supports the argument that traditional variable-centered approaches may obscure critical diversity in responses to job demands and available resources.

This enhanced conceptualization is instrumental in advancing models of occupational health by integrating interpersonal stressors as potent job demands that deplete resources necessary for maintaining high WE levels. Practically, the findings offer a clear roadmap for tailored intervention strategies within public healthcare organizations. For instance, the identification of the *Balanced* profile reflects the typical conditions experienced by HW, while the *Engaged* profile delineates a benchmark for optimal functioning. This suggests that enhancing job resources and minimizing excessive job demands can promote healthier working conditions. Conversely, the profiles featuring high levels of WB, indicate that persistent exposure to WB not only is associated with diminishes WE but also with exacerbates EE, related with reduced performance, lower job satisfaction, deteriorated health outcomes, and an increased intention to quit. These insights imply that interventions should not solely focus on workload management but must also address adverse interpersonal behaviours and foster supportive organizational climates to mitigate the negative spiral of stress and disengagement.

### Conclusion

Our study contributes to the literature by demonstrating that distinct latent profiles – in terms of WB, EE, and WE – capture the nuanced reality of Italian public HW. The *Engaged* profile suggests that when job demands are effectively counterbalanced by appropriate resources, employees present higher satisfaction and job performance. Conversely, profiles marked by simultaneously high WB and EE underscore the detrimental effects of adverse interpersonal experiences on well-being and overall work outcomes.

These findings highlight the importance, as demonstrated by previous research, of not only reducing job demands but also actively fostering both job and personal resources. Therefore, interventions should adopt a dual approach: minimizing excessive demands while simultaneously strengthening resources available to public healthcare settings, to increase the number of employees belonging to the engage profile.

## Data Availability

Data and questionnaires are available upon request from the corresponding author.

## Action editor

Lars-Gunnar Lundh served as action editor for this article.

## Open access

This article is distributed under the terms of the Creative Commons Attribution 4.0 International License (http://creativecommons.org/licenses/by/4.0/), which permits unrestricted use, distribution, and reproduction in any medium, provided you give appropriate credit to the original author(s) and the source, provide a link to the Creative Commons license, and indicate if changes were made.

## Appendix

**Appendix A:** Description of measures included in the study.

Measures	Number of items	Range	Example of item
*Indicators*			
Workplace bullying	9	1 – 5	You were ignored, excluded, or marginalized
Emotional exhaustion	3	1 – 5	At work, I feel mentally exhausted
Work engagement	3	0 – 6	At my work, I feel bursting with energy
*Job demands*			
Quantitative demands	5	1 – 5	How often does your job require you to work very fast?
Perceived injustice	4	1 – 6	Promotion is gained through personal connections
Role conflict	3	1 – 5	Does your work have clear objectives?
*Job resources*			
Quality of work environment	4	1 – 5	The technology and/or other 'tools of the trade' are functioning satisfactorily.
Career development	3	1 – 5	Do you have the possibility of learning new things through your work?
Horizontal and vertical trust	4	1 – 5	Does the management trust the employees to do their work well?
Social support	3	1 – 5	How often do you get help and support from your immediate superior, if needed?
*Job resources*			
Self-efficacy at work	4	1 – 6	Regardless of what happens, I usually manage
Passion at work	6	1 – 7	This activity allows me to live a variety of experiences
*Outcomes*			
Physical symptoms	5	1 – 5	. I suffer from headaches
Psychological symptoms	5	1 – 5	I feel tense and stressed
Intention to quit	3	1 – 5	To quit your job
Performance	6	1 – 5	We achieve our work goals
Job satisfaction	1	1 – 10	On a scale of 1 to 10, how satisfied are you overall with your job?

**Appendix B:** Correlations among the main variables

	*M*	*SD*	*N*	1	2	3	4	5	6	7	8	9	10	11	12	13	14	15	16
1. Workplace bullying	2.15	0.97	624	--															
2. Emotional exhaustion	3.41	1.05	624	.27^**^	--														
3. Work engagement	3.36	1.40	624	-.31^**^	-.36^**^	--													
4. Quantitative demands	3.90	0.90	624	.17^**^	.49^**^	-0.04	--												
5. Perceived injustice	3.70	1.49	599	.38^**^	.22^**^	-.23^**^	.13^**^	--											
6. Role conflict	3.42	1.07	624	.50^**^	.40^**^	-.35^**^	.39^**^	.39^**^	--										
7. Quality of work environment	3.00	0.89	624	-.22^**^	-.29^**^	.37^**^	-.30^**^	-.17^**^	-.42^**^	--									
8. Career development	3.57	1.04	624	-.33^**^	-.21^**^	.44^**^	0.07	-.25^**^	-.32^**^	.36^**^	--								
9. Horizontal and vertical trust	3.03	0.93	624	-.48^**^	-.31^**^	.47^**^	-.16^**^	-.45^**^	-.52^**^	.47^**^	.45^**^	--							
10. Social support	3.46	1.07	624	-.51^**^	-.24^**^	.37^**^	-0.07	-.31^**^	-.40^**^	.32^**^	.41^**^	.59^**^	--						
11. Self-efficacy at work	4.40	0.94	624	-.11^**^	-.17^**^	.49^**^	0.00	0.06	-.10^*^	.28^**^	.27^**^	.28^**^	.17^**^	--					
12. Passion at work	3.99	1.42	624	-.26^**^	-.39^**^	.71^**^	-.12^**^	-.22^**^	-.34^**^	.38^**^	.56^**^	.46^**^	.37^**^	.45^**^	--				
13. Physical symptoms	2.51	0.95	624	.35^**^	.47^**^	-.30^**^	.18^**^	.18^**^	.24^**^	-.18^**^	-.18^**^	-.26^**^	-.25^**^	-.21^**^	-.27^**^	--			
14. Psychological symptoms	2.97	1.01	624	.39^**^	.68^**^	-.36^**^	.32^**^	.21^**^	.34^**^	-.25^**^	-.21^**^	-.36^**^	-.29^**^	-.28^**^	-.38^**^	.65^**^	--		
15. Intention to quit	2.81	1.24	618	.38^**^	.50^**^	-.44^**^	.28^**^	.34^**^	.44^**^	-.32^**^	-.33^**^	-.45^**^	-.37^**^	-.18^**^	-.49^**^	.41^**^	.49^**^	--	
16. Performance	5.03	1.34	618	-.38^**^	-.19^**^	.41^**^	-0.03	-.16^**^	-.29^**^	.33^**^	.34^**^	.48^**^	.55^**^	.32^**^	.39^**^	-.19^**^	-.23^**^	-.31^**^	--
17. Job satisfaction	5.88	2.42	624	-.37^**^	-.47^**^	.71^**^	-.17^**^	-.26^**^	-.38^**^	.42^**^	.52^**^	.50^**^	.40^**^	.38^**^	.76^**^	-.37^**^	-.47^**^	-.60^**^	.40^**^

* *p* < 0.05.

** *p* < 0.01.
